# Persistent mucus plugs in proximal airways are consequential for airflow limitation in asthma

**DOI:** 10.1172/jci.insight.174124

**Published:** 2024-02-08

**Authors:** Brendan K. Huang, Brett M. Elicker, Travis S. Henry, Kimberly G. Kallianos, Lewis D. Hahn, Monica Tang, Franklin Heng, Charles E. McCulloch, Nirav R. Bhakta, Sharmila Majumdar, Jiwoong Choi, Loren C. Denlinger, Sean B. Fain, Annette T. Hastie, Eric A. Hoffman, Elliot Israel, Nizar N. Jarjour, Bruce D. Levy, Dave T. Mauger, Kaharu Sumino, Sally E. Wenzel, Mario Castro, Prescott G. Woodruff, John V. Fahy, for the NHLBI Severe Asthma Research Program (SARP)

**Affiliations:** 1 Division of Pulmonary, Critical Care, Allergy and Sleep Medicine, Department of Medicine, and; 2 Department of Radiology and Biomedical Imaging, UCSF, San Francisco, California, USA.; 3 Department of Radiology, Duke University, Durham, North Carolina, USA.; 4 Department of Radiology, UCSD, San Diego, California, USA.; 5 Cardiovascular Research Institute and; 6 Department of Epidemiology and Biostatistics, UCSF, San Francisco, California, USA.; 7 Division of Pulmonary, Critical Care and Sleep Medicine, University of Kansas School of Medicine, Kansas City, Kansas, USA.; 8 Division of Allergy, Pulmonary, and Critical Care Medicine, University of Wisconsin School of Medicine and Public Health, Madison, Wisconsin, USA.; 9 Department of Radiology, University of Iowa, Iowa City, Iowa, USA.; 10 Department of Internal Medicine, Section for Pulmonary, Critical Care, Allergy and Immunology, Wake Forest School of Medicine, Winston-Salem, North Carolina, USA.; 11 Division of Pulmonary and Critical Care Medicine, Department of Medicine, Brigham and Women’s Hospital, Boston, Massachusetts, USA.; 12 Division of Biostatistics and Bioinformatics, Penn State College of Medicine, The Pennsylvania State University, Hershey, Pennsylvania, USA.; 13 Division of Pulmonary and Critical Care Medicine, Washington University, St. Louis, USA.; 14 Department of Environmental and Occupational Health, University of Pittsburgh, Pittsburgh, Pennsylvania, USA.; 15 The NHLBI SARP centers are listed in Acknowledgements

**Keywords:** Pulmonology, Asthma, Clinical practice, Diagnostic imaging

## Abstract

**BACKGROUND:**

Information about the size, airway location, and longitudinal behavior of mucus plugs in asthma is needed to understand their role in mechanisms of airflow obstruction and to rationally design muco-active treatments.

**METHODS:**

CT lung scans from 57 patients with asthma were analyzed to quantify mucus plug size and airway location, and paired CT scans obtained 3 years apart were analyzed to determine plug behavior over time. Radiologist annotations of mucus plugs were incorporated in an image-processing pipeline to generate size and location information that was related to measures of airflow.

**RESULTS:**

The length distribution of 778 annotated mucus plugs was multimodal, and a 12 mm length defined short (“stubby”, ≤12 mm) and long (“stringy”, >12 mm) plug phenotypes. High mucus plug burden was disproportionately attributable to stringy mucus plugs. Mucus plugs localized predominantly to airway generations 6–9, and 47% of plugs in baseline scans persisted in the same airway for 3 years and fluctuated in length and volume. Mucus plugs in larger proximal generations had greater effects on spirometry measures than plugs in smaller distal generations, and a model of airflow that estimates the increased airway resistance attributable to plugs predicted a greater effect for proximal generations and more numerous mucus plugs.

**CONCLUSION:**

Persistent mucus plugs in proximal airway generations occur in asthma and demonstrate a stochastic process of formation and resolution over time. Proximal airway mucus plugs are consequential for airflow and are in locations amenable to treatment by inhaled muco-active drugs or bronchoscopy.

**TRIAL REGISTRATION:**

Clinicaltrials.gov; NCT01718197, NCT01606826, NCT01750411, NCT01761058, NCT01761630, NCT01716494, and NCT01760915.

**FUNDING:**

AstraZeneca, Boehringer-Ingelheim, Genentech, GlaxoSmithKline, Sanofi–Genzyme–Regeneron, and TEVA provided financial support for study activities at the Coordinating and Clinical Centers beyond the third year of patient follow-up. These companies had no role in study design or data analysis, and the only restriction on the funds was that they be used to support the SARP initiative.

## Abstract 2

<p>Persistent mucus plugs in proximal airway generations occur in asthma, are consequential for airflow, and are in airway locations amenable to treatment.</p>

## Graphical abstract

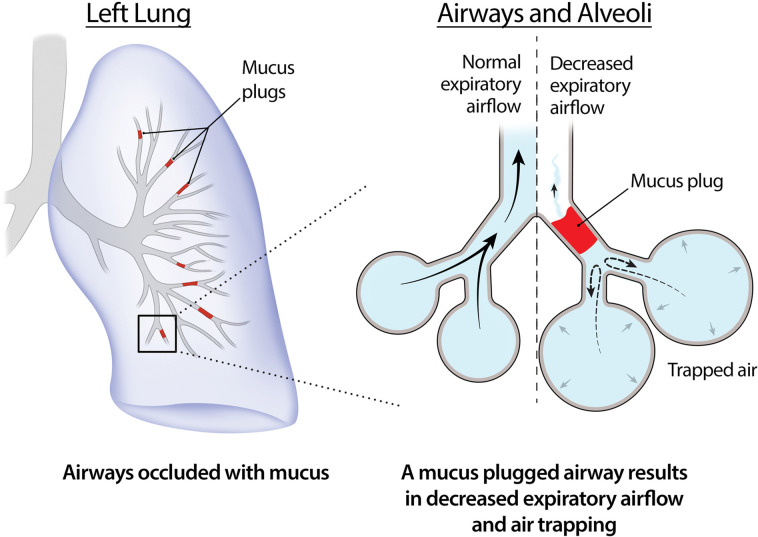

## Introduction

Severe forms of asthma are characterized by airflow obstruction that does not always normalize with treatments that target excessive airway smooth muscle tone or airway inflammation (1). Persistent airway mucus plugs are prevalent in severe forms of asthma and represent a plausible mechanism of chronic airflow obstruction in these patients (2, 3). In addition, mucus plugs in chronic obstructive pulmonary disease are associated with more severe airflow obstruction (4) and increased risk of mortality (5). Furthermore, mucus plugs occur in patients taking corticosteroid treatment (2), indicating that treatment of inflammation is not sufficient to prevent formation of these plugs. Specific treatments of mucus plugs could involve drugs to decrease the formation of new mucus plugs, drugs to remove existing plugs, or mechanical approaches such as mucus clearance devices or bronchoscopy. The rational development or selection of best treatments to remove mucus plugs requires quantitative data about their structural features and airway tree location, but this information is currently lacking.

Measuring the size and shape of mucus plugs requires volumetric information. Analogous to methods to quantify the 3-dimensional (3D) geometry of solid tumors in the lung (6), the 3D geometry of airway mucus plugs can be reconstructed from sequences of 2D CT lung images. In addition, the location of mucus plugs in the airway tree can be determined using methods of airway segmentation (7). The use of these image-based methods to study the physiological consequences of mucus plugs is feasible in the SARP-3 program because the deep phenotyping protocol in SARP-3 includes repeated CT lung scans and detailed lung function measures (8). Our overarching goal for this study was to characterize the size, shape, and location of mucus plugs in patients with asthma over time and to determine how these mucus plug features influence airflow obstruction and air trapping.

## Results

### Annotations of mucus plugs in CT lung scans provide potentially novel measures of airway mucus plug burden.

**Figure 1:**
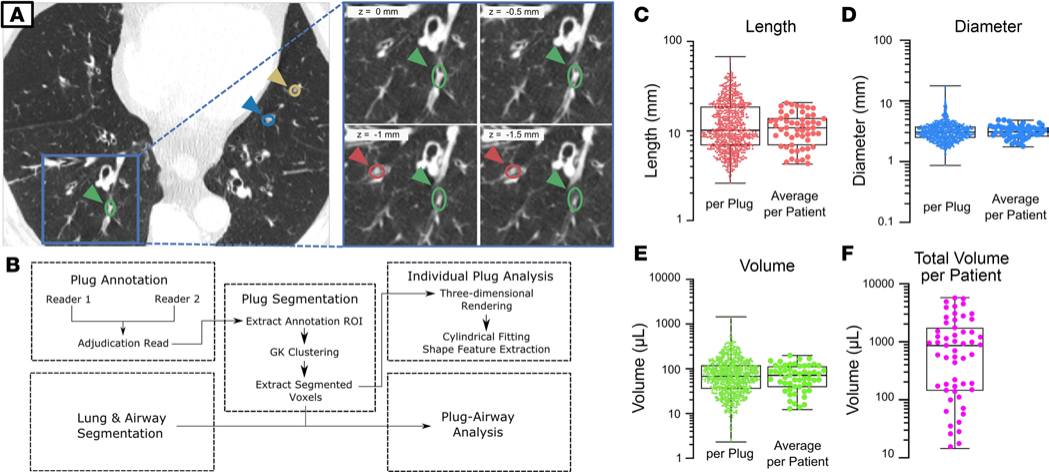
Mucus plugs are heterogeneous in size and shape in asthma. (**A** and **B**) Overview of annotation and image processing pipeline for mucus plug shapes quantification. (**A**) An elliptical mark placed over each plug generates a center coordinate, width, and height for a region of interest (colored arrows). The process is repeated at each axial slice (inset), with *z* indicating the slice location relative to the initial image and with color denoting annotations belonging to the same plug. (**B**) Annotations are incorporated into an image-processing pipeline for segmentation and feature extraction on plugs, enabling calculation of their length, diameter, and volume. (**C**–**E**) Results of shape feature quantification of individual mucus plugs (left, *n* = 778 plugs) and averages by patient (right, *n* = 55 patients) from baseline scans, including plug length (**C**), plug diameter (**D**), and plug volume (**E**). Note that scales are logarithmic. Bars indicated interquartile range, and whiskers show minimum and maximum values. (**F**) Total mucus volume per patient. GK, Gustafson-Kessel.

A previously described mucus plug “segment score” (Supplemental Figure 1A; supplemental material available online with this article; https://doi.org/10.1172/jci.insight.174124DS1) is generated when a radiologist assigns a point to each bronchopulmonary segment in a CT lung scan that has at least 1 airway occluded by mucus (2). Although of proven utility (2–4), the segment score has a limited range of values (values 1–20), is not fully quantitative, and does not provide information about the airway location of a mucus plug or its shape and size features. To address these limitations and answer research questions related to mucus plug characterization, we optimized methods in which annotators (thoracic radiologists) used a Digital Imaging and Communications in Medicine (DICOM) viewer to place elliptical markings on airways occluded by mucus in 2D axial slices of CT lung scans (Figure 1A and Supplemental Video 1). A clustering algorithm (9) applied to these elliptical annotations allowed the plugs to be segmented, reconstructed in 3D, and enumerated (Figure 1B). In this way, thoracic radiologists generated 12,476 unique annotations related to 778 individual whole mucus plugs in CT scans from 57 patients with asthma, whose clinical characteristics are shown in Table 1. By assigning a point for each elliptical annotation within a patient’s CT lung scan, a patient-specific “mucus slice score” could be calculated from the sum of these points (Supplemental Figure 1B). The mucus slice scores correlate with the mucus plug segment scores (Supplemental Figure 1D) but provide more quantitative information and a larger range of values. The total number of discrete mucus plugs per patient, which we denote as the “mucus plug score,” is another total mucus plug burden score with similar advantages (Supplemental Figure 1, C and E).

### Mucus plugs are heterogeneous in size and cluster into “stubby” and “stringy” phenotypes.

To quantify the shapes and sizes of mucus plugs, the voxels for each mucus plug were extracted (Supplemental Video 2), and the size of each plug was computed and quantified. We found that the length, diameter, and volume of individual mucus plugs varied across 1 or more orders of magnitude (Figure 1, C–E, and Table 2), indicating a high degree of heterogeneity in the size of mucus plugs in asthma. To quantify the volume of mucus plugs within each patient, we generated a total mucus volume measure, which also varied by multiple orders of magnitude (Figure 1F).

**Figure 2:**
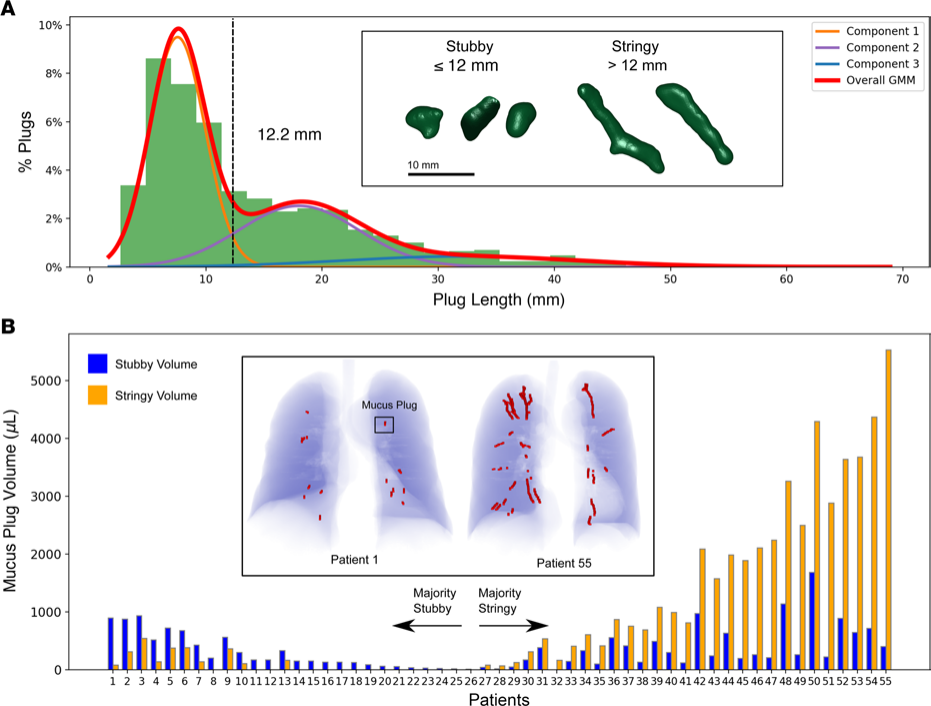
Mucus plugs exhibit multiple underlying length phenotypes. (**A**) Distribution of mucus plug lengths with best-fit Gaussian mixture model by Akaike information criterion, with separation between short (“stubby”) plugs and long (“stringy”) plugs at 12 mm. Very long plugs (component 3) make up a small portion of total population. The inset shows 3D renderings of stubby and stringy mucus plugs. (**B**) Distribution of mucus plug volume in each patient (*n* = 55) ordered by predominance of stubby versus stringy mucus plugs within each patient. The inset image provides renderings of mucus plugs (red) within the lung of a patient with a majority stubby plug volume (patient 1) and majority stringy plug volume (patient 55).

The distribution of mucus plug lengths appeared to be multimodal (Figure 2A), and assessment of model fit by Akaike information criterion revealed that a Gaussian mixture model with 3 underlying distributions had the highest likelihood (Supplemental Figure 2). Based on a length of 12 mm separating the 2 dominant populations in the model, we defined 2 plug phenotypes based on length — short plugs that were 12 mm or less in length, denoted as “stubby,” and long plugs that were more than 12 mm in length, denoted as “stringy.” In this way, we found that, among 778 plugs, 448 were stubby and 330 were stringy (example renderings are shown in Figure 2A). Information on the numbers of stubby and stringy mucus plugs within each patient allowed determination of the mucus plug volume in each patient attributable to stubby versus stringy plugs. As shown in Figure 2B, the patients with the highest total mucus volumes achieved these levels mainly because of volume contributed by stringy mucus plugs.

Because eosinophilic inflammation — eosinophil counts and levels of eosinophil peroxidase (EPX) in blood and sputum — are known to be linked to mucus plug segment scores in asthma (2), we explored whether the size of individual mucus plugs was influenced by eosinophilic inflammation. We found that the average mucus plug length and volume in patients were positively correlated with blood eosinophil counts and sputum EPX levels (Supplemental Figure 3, A–D).

### Mucus plugs in CT lung images primarily localize to airways that are 2–4 mm in diameter.

**Figure 3:**
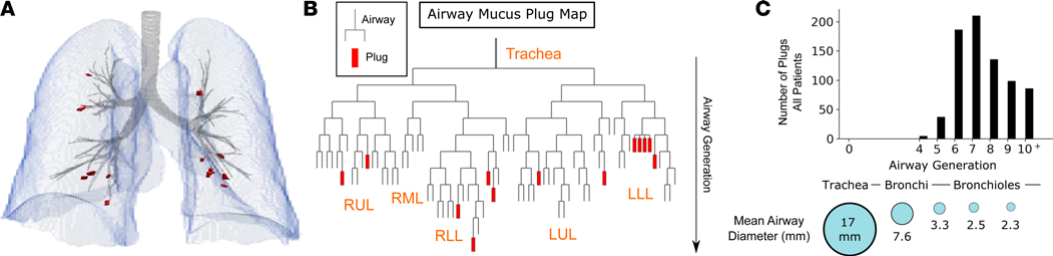
Mucus plugs are primarily located in proximal airway generations in asthma. (**A**) Rendering of segmented lung parenchyma (blue), airways (gray), and mucus plugs (red) in a patient with asthma. (**B**) Mucus plug map showing topological location of mucus plugs in the airway for the same patient. Generation number is counted by each airway bifurcation with trachea as generation 0. (**C**) Histogram showing that mucus plugs are located primarily in airway generations 6–9, which have a diameter of 2–4 mm. Data are from 778 plugs visible in 55 baseline CT lung scans, and the mean airway diameter is the average diameter measured at each airway generation across the 55 scans.

By segmenting lung parenchyma and airways on a lobar basis, every mucus plug could be localized to a specific airway branch and lobe (Figure 3A). This information allowed the creation of a patient-specific “airway mucus plug map,” a visualization of the location of each mucus plug within the branching airway tree (Figure 3B). To summarize the airway generations occluded by all 778 mucus plugs, we generated a frequency distribution plot that shows that mucus plugs are located primarily in generations 6, 7, 8, and 9 (Figure 3C). We estimated these airways to be typically 2–4 mm in diameter in the CT lung scans analyzed (Figure 3C). We explored whether there was a specific pattern of length or volume of individual mucus plugs in different airway generations but did not find any trend (Supplemental Figure 4, A and B). Although the number of mucus plugs did not differ significantly in upper versus lower lobes or in the right versus the left lung, the volumes of individual mucus plugs in the lower lobes were greater than the volumes of individual mucus plugs in the upper and middle lobes (Supplemental Figure 4C).

### Mucus plugs persist in the same airways for many years but demonstrate dynamic changes in size over time.

**Figure 4:**
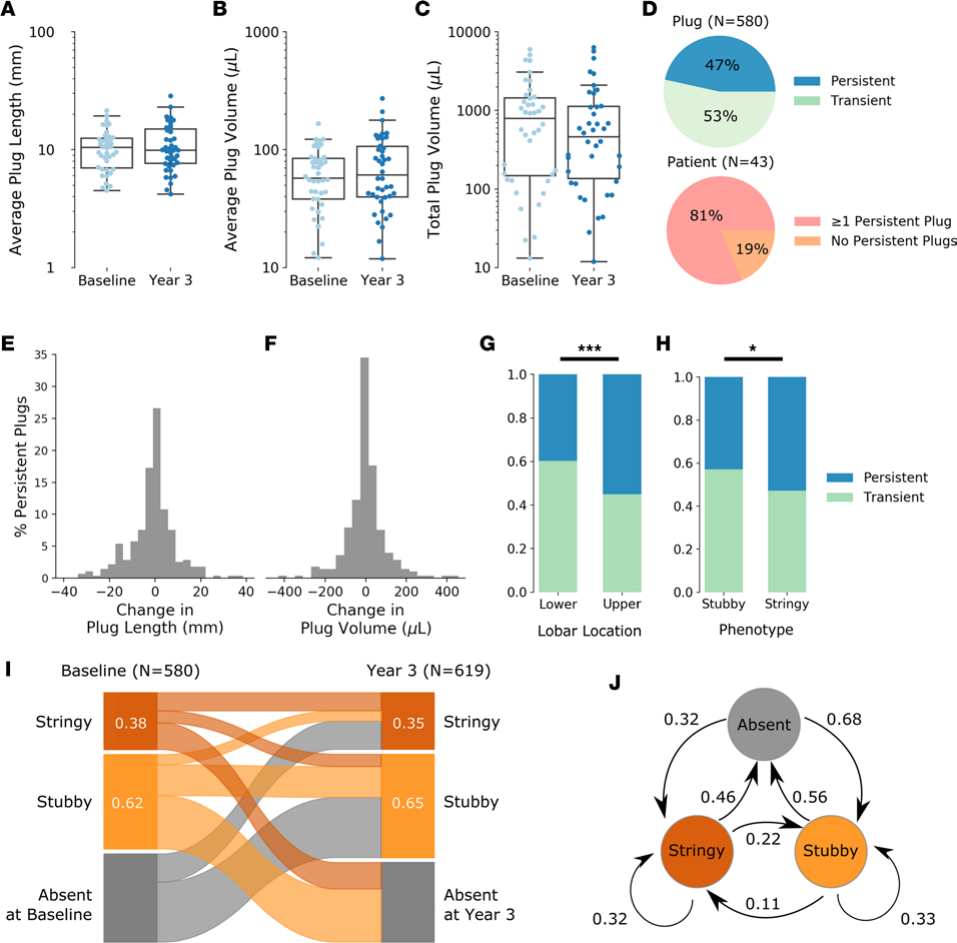
Mucus plugs persist for years in the same airways and demonstrate dynamic changes in size over time. (**A**–**C**) Comparison of patient-level measurements for patients with matched baseline and year-3 scans with average plug length per patient (**A**), average plug volume per patient (**B**), and total plug volume per patient (**C**) all showing similar between baseline and year 3. Data in **A**–**C** are from 580 baseline plugs and 619 year-3 plugs visible in 86 CT lung scans from 43 patients. Bars indicate interquartile range (IQR), and whiskers indicate 1.5 IQR. (**D**) Percentage of individual plugs classified as persistent or transient using analyses of scans at baseline and year 3 (upper pie chart). Percentage of patients with at least 1 persistent plug in the same airway at baseline and at year 3 (lower pie chart). (**E**) Frequency distribution plot showing the change in mucus plug length from baseline to year 3. (**F**) Frequency distribution plot showing the change in mucus plug volume from baseline to year 3. Data in **E** and **F** are from 270 persistent mucus plugs. (**G**) Compared with mucus plugs in lower lobe locations, mucus plugs in upper lobe locations are more likely to persist for 3 years. ***P < 0.001 (Kruskal-Wallis test). (**H**) Compared with stubby plugs, stringy mucus plugs are more likely to persist for 3 years. *P < 0.05 (Kruskal-Wallis test). (**I**) Sankey plot showing how stringy, stubby, and absent mucus plug phenotypes vary from baseline to year 3. (**J**) State-transition diagram showing the probability of transition between stubby, stringy, and absent plug group from baseline to year 3.

Of the 57 patients whose baseline CT lung scans were annotated, 43 had a second CT lung scan available at their year-3 visit that allowed analysis of mucus plugs over time. Among scans from the 43 patients, 580 mucus plugs were visible on the baseline scans and 619 mucus plugs were visible on the year-3 scans. We found that the per-patient average plug length, average plug volume, and total mucus plug volume did not differ significantly between baseline and year 3 (Figure 4, A–C), indicating overall stability of total mucus plug burden within patients over 3 years. To explore the temporal dynamics of the 580 mucus plugs identified in the baseline scans from the 43 patients, we tracked mucus plugs that persisted in the same airway between the baseline and year-3 scans (Supplemental Video 3), labeling these plugs as “persistent.” We also tracked mucus plugs that disappeared between the baseline and year-3 scans, labeling these plugs as “transient.” Remarkably, we found that 47% of the 580 baseline plugs persisted in the same airway for 3 years, and 81% of the 43 patients had at least 1 persistent plug (Figure 4D). Persistent mucus plugs, although static in location, exhibited dynamic behavior in size and underwent variable changes in length and volume (Figure 4, E and F). Changes were centered around zero and appeared normally distributed (Supplemental Figure 5), and there was no statistically significant difference in average length or volume of the entire population of plugs over the 3-year period. In addition, the finding that the total mucus volume per patient stayed, on average, constant over time (Figure 4C) was consistent with the observation that the disappearance of transient mucus plugs sometimes coincided with the appearance of new mucus plugs in different airways at year 3.

In comparing the characteristics of persistent and transient mucus plugs, we found that persistent mucus plugs were longer, more frequently stringy, and more frequently located in the upper lobes (Table 3 and Figure 4, G and H). We analyzed the CT attenuation of the pixels in each plug by computing the median value in Hounsfield units (HU) and found that transient plugs were more radiodense (Table 3). In analyzing the 3-year behavior of stringy versus stubby plugs using Sankey plot and state-transition analyses, we found that, among plugs that persisted, stubby plugs were more likely to stay stubby and stringy plugs were more likely to stay stringy (Figure 4, I and J).

### Mucus plugs in proximal airways have larger effects on spirometric measures of lung function than plugs in distal airways.

**Figure 5:**
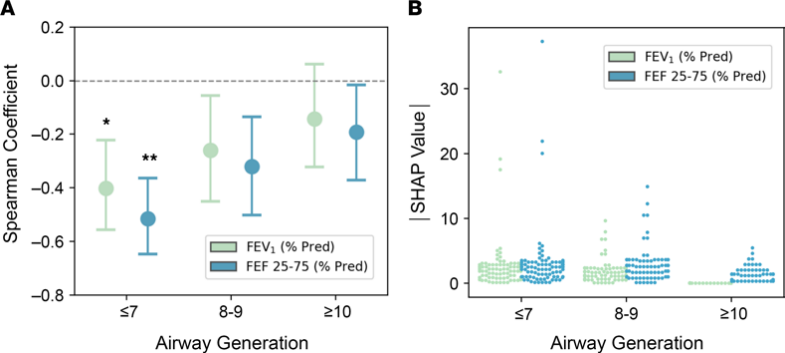
Proximal plugs are more consequential for spirometric measures of airflow obstruction in asthma. (**A**) Correlation analysis of mucus plugs grouped independently by proximal airway generations (7 or less), intermediate airway generations (8 and 9), and distal airway generations (10 and greater) as well as spirometry measures. The estimated Spearman coefficients for each generation group, which correlate plug count by generation group with FEV_1_ and FEF_25–75_, are shown for proximal, intermediate, and distal airway generations along with 95% CI. *P < 0.05, different from distal airway generations for FEV_1_ (by bootstrapping). **P < 0.01, different from distal airway generation (by bootstrapping). Data presented includes pooled baseline and year-3 follow-up scans (*n* = 97). (**B**) Absolute SHAP values for FEV_1_ and FEF_25–75_ at proximal, intermediate, and distal airway generations (*n* = 97).

Consistent with our previously reported results (2, 3), overall mucus plug burden as assessed by mucus segment score, mucus plug score, and mucus slice score was inversely associated with forced expiratory volume in 1 second (FEV_1_) (Supplemental Figure 6). Our localization of mucus plugging to specific airway branches, however, allowed us to compare the relative effects of mucus plugs in proximal airways (generations 7 or less), intermediate airways (generations 8 and 9), and distal airways (generation 10 and greater). We used correlation coefficients and SHapley Additive exPlanation (SHAP) values (which consider plug count in each generation as an independent feature in a linear regression) to compare the relative effects of mucus plug count in proximal, intermediate, and distal airways on spirometric measures of airflow. In these analyses, the mucus plugs were grouped independently by airway generation for each patient, and the number of mucus plugs per generation was counted for each patient. The plug count by generation was correlated with spirometry, either the postbronchodilator FEV_1_ or the forced expiratory flow between 25% and 75% of forced vital capacity (FEF_25–75_), to estimate a Spearman coefficient. For these analyses, the CT scans and lung physiology data from the baseline and year-3 visits were pooled so that 97 CT scans from 57 patients were analyzed. We found that the correlation coefficients (*r*_s_) for mucus plugs in proximal airways (generation ≤ 7) and FEV_1_ or FEF_25–75_ were more negative than the coefficients for mucus plugs and FEV_1_ or FEF_25–75_ in distal airways (generation ≥ 10; Figure 5A), indicating a stronger negative effect of those plugs on airflow. In addition, the magnitude of SHAP values for mucus plugs in proximal airways was larger than those in distal airways (Figure 5B), also indicating a stronger effect from proximal plugs.

### Mucus plugs are associated with airway-specific increases in resistance score and air trapping.

**Figure 6:**
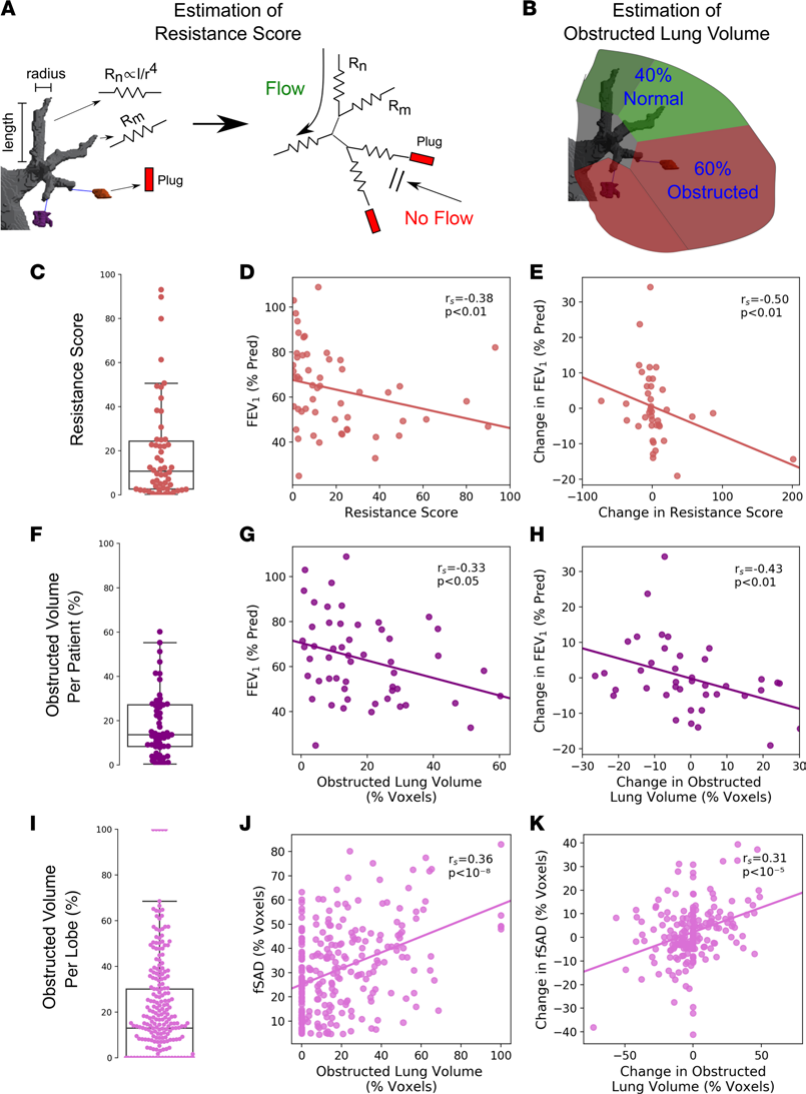
Mucus plugs are associated with an increase in modeled airway resistance and in measured air trapping in lung regions distal to mucus-occluded airways. (**A**) Schematic illustrating computation of resistance score (RS) by incorporating mucus plugging into the airway tree. The airway tree is divided into different segments with an effective resistance *R_n_* given by the length and radius of the airway at that location. After combining all segments, the net resistance of the airway tree in the presence of plugging (*R^p^*) is compared with the resistance of the native airway tree in the absence of plugs (*R^a^*) to yield the increased percentage in airway resistance RS = (100 × [*R^p^ – R^a^*]/R^a^) due to plugs. (**B**) Estimation of obstructed lung volume percentage (OLVP). The voxel volume of the lung region distal to a mucus occluded airway (V^o^) was divided by the total voxel volume in the lobe (V^t^) to generate the estimated obstructed lung volume percentage (100 × V^o^/V^t^). (**C**) Distribution of RS for patients at baseline (*n* = 54). (**D**) Relationship between predicted RS and FEV_1_ at baseline. (**E**) Relationship between changes in predicted RS and changes in FEV_1_ over 3 years for matched patients (*n* = 40). (**F**) Distribution of OLVP per patient at baseline (*n* = 53). (**G**) Relationship between OLVP and FEV_1_ at baseline. (**H**) Relationship between changes in predicted OLVP and changes in FEV_1_ over 3 years for matched patients (*n* = 40). Sensitivity analysis of outlier point (ΔRS = 201, ΔFEV_1_ = –14%) shows similar correlation coefficient (*r*_s_ = –0.50, *P* = 0.001 with outlier included and *r*_s_ = –0.46, *P* = 0.003 with outlier excluded). (**I**) Distribution of OLVP per lobe at baseline (*n* = 260). (**J**) Relationship between OLVP and disease probability measure functional small airways disease (DPM-fSAD) per lobe at baseline. (**K**) Relationship between changes in OLVP and DPM-fSAD per lobe over 3 years (*n* = 195). *r*_s_ denotes Spearman correlation coefficient. Statistical results of linear mixed model and multivariate regression are shown in Table 4.

We hypothesized that mucus plugs occlude airways, causing airflow obstruction in the conducting airway tree and air trapping in the lung parenchyma distal to affected airways. To explore this hypothesis, we developed simplified models of airflow and air trapping that explicitly incorporate mucus plugs as obstructing airflow in plugged airways. These patient-specific models intake the segmented airways, lungs, and mucus plugs for each individual CT scan and output 2 measures: (a) the resistance score (RS), an estimated effect on the large airway resistance due to mucus plugs (Figure 6A), and (b) the obstructed lung volume percentage (OLVP), an estimate of percentage of lung parenchyma distal to airways occluded by mucus plugs and likely to exhibit air trapping (Figure 6B). Consistent with wide variation in total mucus plug burden between patients (Figure 1F), we found that RS and OLVP values also varied widely between patients (Figure 6, C and F). In cross-sectional analyses of data from the baseline CT lung scans, both values showed significant inverse associations with FEV_1_ (Figure 6, D and G) and FEF_25–75_ (Supplemental Figure 7, A and C). In addition, the changes in RS and in OLVP from baseline to year 3 correlated with changes in FEV_1_ (Figure 6, E and H) and FEF_25–75_ (Supplemental Figure 7, B and D). For the analyses in Figure 6E, we performed a sensitivity analysis to determine the effects of an outlier with ΔRS of 201 and ΔFEV_1_ of –14%. We found that the *r*_s_ was –0.50 (*P* = 0.001) with this outlier included and –0.46 (*P* = 0.003) with the outlier excluded.

Our air trapping model posits that air trapping is spatially associated with occluded airway branches. To test this assumption, we generated lung lobe-specific data for OLVP (Figure 6I) and analyzed the relationship between OLVP and the disease probability measure of functional small airway disease (DPM-fSAD), a previously described measure of air trapping (10). DPM-fSAD is quantified from CT lung scans by registering images acquired at inspiration to images acquired at expiration and, on a voxel-by-voxel basis, identifying regions of the lung that trap gas (10). We found that lobe-specific OLVP measures correlated significantly with fSAD at baseline (Figure 6J) and that the change in lobe-specific OLVP from baseline to year 3 correlated with changes in fSAD (Figure 6K). OLVP also significantly correlated with 2 other CT-based functional measures related to air trapping, (a) the Jacobian mean (the inspiratory to expiratory local lung volume ratio) and (b) expiratory low attenuation area percent below –856 HU (LAA^856^%), on a lobar basis (Supplemental Figure 8). Analysis from linear mixed-effects models to control for multiple measurements from the same patient as well as multivariate regression controlling for age, BMI, sex, and airway wall thickness (covariates determined by our directed acyclic graph in Supplemental Figure 9) were consistent with these results (Table 4). In particular, all measures relating OLVP to measures of airflow and air trapping remained statistically significant when controlling for all covariates. Taken together, these data support the interpretation that mucus plugs specifically cause air trapping in the lung region distal to the airways they occlude.

**Figure 7:**
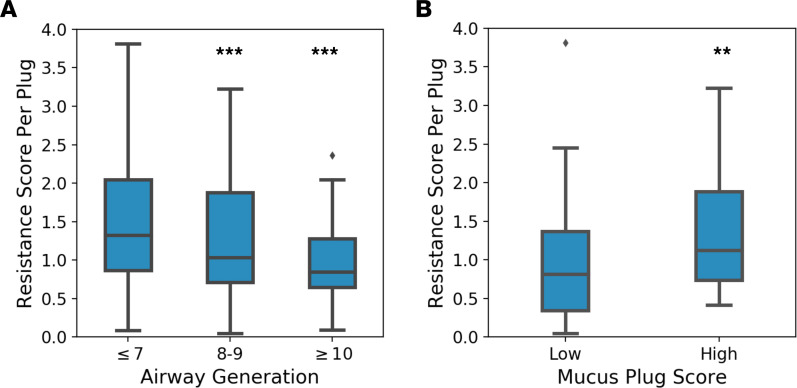
Mucus plugs in proximal generations have a great effect on resistance score than distal generations. (**A**) Resistance score (RS) per plug grouped independently by proximal airway generations (7 or less), intermediate airway generations (8 and 9), and distal airway generations (10 and greater) (*n* = 1,327 plugs). The RS values for each generation group are shown for proximal, intermediate, and distal airway generations. ***P < 0.001 by Kruskal-Wallis test; *P* = 0.008 and *P* = 0.002 for comparison with proximal versus intermediate and proximal versus distal generations, respectively, using linear mixed model with random effects for patient. (**B**) RS per plug grouped independently by mucus plug score-high (>11 plugs) and plug score-low (≤11 plugs) (*n* = 94 patients). **P < 0.01 by Kruskal-Wallis test; *P* = 0.014 for linear mixed model with random effects for patient.

We next used the RS to further test if mucus plugs located in more proximal locations are more consequential for airflow obstruction. For this analysis, we calculated the RS in each patient divided by mucus plug score (i.e., plug count) to estimate RS per plug as a measure of each individual plug’s effect on airflow obstruction. We stratified mucus plugs by proximal (generation ≤ 7), intermediate (generation 8–9), and distal (generation ≥ 10) airway generation and found that plugs in proximal generations had significantly higher RS per plug than intermediate or distal generations (Figure 7A). We similarly stratified plugs from patients with high and low mucus plug scores based on the median value of baseline patients, 11 plugs. We found that plugs in patients with high mucus plug scores had a higher RS-per-plug score (Figure 7B), consistent with the interpretation that, as mucus plugs begin to occlude a substantial fraction of large airways and leave fewer airways patent, subsequent mucus plugs have a higher marginal effect on net airway resistance. These data support our hypothesis that more numerous mucus plugs in more proximal locations are more consequential for airflow obstruction and air trapping than sparser and more distal mucus plugs.

### Quantitative assessment of airway mucus plug pathology.

The analysis of mucus plugs in CT lung scans in asthma presented above yields multiple potentially novel quantitative measures of mucus plug pathology in the lung. Since these measures may serve as biomarkers of mucus pathology, we have summarized them as the Quantitative Assessment of Airway Mucus Plug Pathology (qAAMP) in Table 5. All of the qAAMP measures can be generated in CT lung scans using the workflow described above and in Methods.

## Discussion

Previous studies of the size features of mucus plugs in asthma and their location in the airway tree have relied on analyses of mucus plugs in lung tissues from cases of fatal asthma (11) or of mucus plugs extracted from the lungs using bronchoscopy (12). These studies have analyzed limited numbers of mucus plugs from small numbers of patients and have been unable to assess the effect of mucus plugs on lung function. Here we have provided detailed size and shape information on 1,397 mucus plugs in 57 patients with asthma, and we identified the airway tree locations occluded by these plugs and their lung function consequences. We show that radiographically visible mucus plugs in asthma were heterogeneous in their size and shape, are located primarily in 2 to 4 mm airways, and persist for many years, often in the same airway. Our modeling data also indicate that mucus plugs increase airway resistance and air trapping in lung regions distal to mucus-occluded airways and provide strong rationale to treat mucus plugs as a strategy to improve airflow in asthma.

We found that the length distribution of mucus plugs in asthma is multimodal, and best fit modeling showed that a plug length of 12 mm defines short (“stubby”) and long (“stringy”) plug phenotypes. Although only 40% of the mucus plugs were stringy, these plugs contributed the most mucus volume in patients with the highest mucus burden. The heterogeneity we describe for the number and size of mucus plugs has great relevance for the design of clinical trials that test interventions to treat mucus plugs. For example, it is likely that more numerous mucus plugs or plugs with a stringy phenotype will take longer to respond to treatment (especially inhaled treatments) than less numerous or stubby plugs. In addition, our 3-year longitudinal data inform thinking about the required duration of mucus plug treatments. We show some cases where the same airway location has persistent plugging for 3 years and other cases where mucus plugs disappear from an airway over time or form in a new airway location (Supplemental Figure 10). Based on our observation that the average plug length and volume in these airways is centered around zero and have a normally distributed change in length and volume, we infer that these plugs persist in the airways and undergo a stochastic process of formation and resolution. These observations indicate that many patients with asthma have a persistent mucus plug phenotype that results from a dynamic balance of mucus plug persistence, resolution, and new formation. Our data give insight into the natural kinetic processes of airway mucus plugs and suggest that, while one-time removal of mucus plugs may have clinical benefit, repeated treatments may be needed to prevent formation of newly formed plugs in susceptible airways.

Prior work in postmortem autopsies in fatal asthma has emphasized the presence of mucus plugs in airways less than 2 mm in diameter, which are typically twelfth generation and smaller in the branching airway tree (13). Our lung image–based approach shows that mucus plugs in asthma also occur in airways that are 2–4 mm in diameter, and these airways include the fourth- and fifth-generation airways that aerate the proximal portions of bronchopulmonary segments. This finding that mucus plugs in asthma occur in segmental and larger subsegmental airways is important because they are likely to have larger effects on lung function in these proximal airway locations. Indeed, compared with mucus plugs in more distal airway locations, we show that mucus plugs in proximal airway locations are more consequential for spirometry-based measures of lung function and model-based estimates of airway resistance. Removal of these mucus plugs is, therefore, a rational strategy to improve lung function in asthma. In this context, our modeling of airway resistance, which is computed by comparing the resistance of the airway tree in the presence and absence of mucus plugs, can be thought of as a “virtual plug extraction.” Our virtual plug extraction data support removal of mucus plugs as a strategy to improve lung function in asthma.

Development of muco-active drugs for lung disease has been slowed by lack of predictive and monitoring biomarkers and by limited information about mucus plug phenotypes to guide drug dosing and formulation. We propose that the qAAMP metrics provided here will have great utility to select patients with mucus plug–high phenotypes for clinical trials of muco-active drugs and to monitor the effects of treatment on mucus plugs in these patients. For example, the qAAMP measures will allow determination of whether a muco-active treatment affects total mucus plug burden and whether this occurs globally in the airway tree or is restricted to specific locations in the airway tree. In terms of guiding drug dosing and drug formulation, the mucus plug volume data will be useful in calculating the delivered drug dose required to lyse mucus plugs. In addition, the airway mucus plug map data and 3D visualizations of the location of persistent plugs (Supplemental Figure 10) will guide optimization of the physiochemical properties of aerosols or mechanical interventions needed to reach mucus plugs in fourth- to tenth-generation airways.

We note 2 limitations of the current study. First, our assessment of airway mucus plugs is limited by the resolution of CT lung scans. This means that our data do not include information about mucus plugs in small airways. Despite this limitation, our data for mucus plugs in larger airways emphasize the presence of plugs in these airways and demonstrate the consequences of these plugs for lung function. Second, the process of generating annotations is time intensive and requires expertise by specialty-trained thoracic radiologists. Prior work has shown promising results in automating plug segmentation using deep learning (14), and the volumetric segmentation data generated here can be used to train analogous algorithms in the asthma population.

In summary, heterogeneously sized mucus plugs in asthma persist for many years and show dynamic changes in their shape and size over time. These mucus plugs in proximal airway locations affect lung function, and they are amendable to treatment by aerosolized drugs or by interventional bronchoscopy. Treatments to remove mucus plugs and prevent their reformation in severe asthma constitutes a rational strategy to improve airflow obstruction in treatment-refractory disease.

## Methods

### Patients.

Patient data were obtained from the NHLBI SARP database, a multiinstitutional cohort designed to obtain longitudinal clinical, serologic, physiologic, and imaging data of patients with severe asthma (15). CT scans were acquired after use of a bronchodilator using a previously described protocol (16). A sample size of 54 was calculated based on an initial power estimate needed to demonstrate an association between mucus plugs in proximal generations and FEV_1_. Based on this estimate, we selected 57 patients from a larger cohort of patients whose CT lung scans had previously been scored by radiologists and shown to have mucus plugs (3). Of the 57 patients, 43 had a second CT lung at year 3. Scans were included in the study reported here if they had at least 1 mucus plug either at baseline or year 3. In total, at the baseline visit, CT scans from 55 patients had mucus plugs that were analyzed and included in the baseline data set; at the year-3 visit, CT scans from 42 patients had mucus plugs that were analyzed and included in the year-3 data set. Scans were excluded if they demonstrated radiographic evidence of active infections, allergic bronchopulmonary aspergillosis, lung scarring, or motion degradation limiting the ability to evaluate for mucus plugs. All eligible SARP-3 scans acquired at the UCSF center were included in the study, and additional scans were randomly sampled from the remainder of the SARP-3 CT lung imaging database.

### Mucus plug annotations.

Thoracic radiologists annotated the chest CTs in this study. The annotation process is illustrated in Figure 1A. Readers used a DICOM viewer (OsiriX; Pixmeo) to place an elliptical marking over each mucus plug within an axial slice. Per previous protocol (17), window width was 1,200 HU and window center was 600 HU during visualization. Voxel spacing of the reconstructed volumes ranged from 0.5 to 0.7 mm in the axial (*x* and *y* axes) plane, and spacing between axial slices (*z* axis) ranged from 0.5 to 0.6 mm. Each annotation yielded a center coordinate, width, and height for a region of interest (ROI) containing the plug at that slice. This process was repeated for every plug and every axial slice in the scan (Figure 1A, inset). Annotations that belonged to a single contiguous plug were designated with a single numerical label.

The annotation process was performed independently twice by 2 radiologists for each scan (Figure 1B). Plugs that were identified by only 1 of 2 readers were considered discordant and reviewed by a third reader for adjudication. Supplemental Video 1 shows the annotations in a CT scan resulting from the 3-reader adjudication process. From the finalized annotation, the mucus segment score was calculated after manual identification of the bronchopulmonary segment containing each mucus plug. The mucus slice score was calculated as the sum of the number of elliptical annotations, and mucus plug score was calculated as the sum of the number of individual mucus plugs.

### Annotation of year 3 scans.

Annotations of the year-3 scans occurred after the baseline scans, and the radiologists had access to the finalized baseline scans results during annotation. Similar to the baseline scan process, 2 radiologists independently annotated each year-3 scan, followed by adjudication by a third radiologist. In certain cases, the annotator of the year-3 scan identified a likely plug on the baseline scan that had not been annotated during the initial process. These possible baseline plugs were collectively reviewed by the entire team of 4 radiologists, and a consensus vote was taken to determine if the plug should be retroactively annotated on the baseline scan. In this manner, an additional 34 plugs in the baseline cohort were identified and annotated. This consensus read was undertaken to obtain higher fidelity data in mucus plug tracking (Figure 4) and in identifying mucus plug persistence over time.

### Mucus plug segmentation, quantification, and visualization.

To segment and analyze individual plugs, we developed a custom computational workflow to ingest and process annotations (Figure 1B). Each annotation was first used to extract an elliptical ROI surrounding each mucus plug in a particular slice. The extracted voxels from all slices belonging to a single mucus plug were combined into a single volumetric subset. A fuzzy clustering algorithm known as Gustafson-Kessel (GK) clustering was used to segment the mucus plug from surrounding lung parenchyma and airway lumen in a manner similar to that described for segmentation of lung nodules (9). In our pipeline, the GK clustering algorithm was run on the extracted volumetric subset and was used to separate voxels into 2 clusters based on imaging intensity (radiodensity). The foreground was taken to be the cluster with the highest intensity value. The single largest contiguous foreground region by volume was then selected as the mucus plug. Results of an example plug segmentation are shown in Supplemental Video 1.

Once individual mucus plugs were segmented on a volumetric basis, their size was estimated using voxel and mesh-based methods (18). The length of each plug was computed by employing principal component analysis on the ROI to calculate eigenvalues along the 3 principal axes (λ_maj_ > λ_min_ > λ_least_) and estimating the length *L* by the following formula: *L* = 4 √ λ_maj_ (18)_._ The diameter was calculated by fitting the 3D mucus ROI to a cylinder and using the resultant best-fit value for the cylinder diameter (19). The CT radiodensity of segmented pixels was analyzed per plug to compute the median density value for each individual plug. For visualization of individual mucus plugs, a triangular mesh representing the surface of the mucus plugs was generated using the marching cubes algorithm (9) with an additional surface smoothing algorithm (20) applied prior to rendering (Figure 2A and Supplemental Video 2).

### Lung and airway segmentation and skeletonization.

Lung parenchyma was segmented on a lobar basis using previously described methods (21) with software available in an open source software package (22). Airway segmentation was performed by combining a region-growing method (22), which yields an estimate of central airways, with a convolutional neural network–based approach (23), which has improved performance in smaller airways. The segmented airway was taken to be the largest contiguous region resulting from the voxel-wise union of the 2 methods. The airway tree was then skeletonized (22) yielding a centerline estimation of the airway tree. A topological representation of the airway tree was generated that contained information for each portion of the airway tree, including the location of centerline points, branching points, length of each segment, local airway radius estimates, airway generation number, and lobar location as well as information about connectivity to more distal (child) branches. Airway termination points were defined as the most distal points of the centerline that no longer had child branches.

### Airway mucus plug map generation.

After individual mucus plugs were segmented, each plug was then localized to a position in the airway tree. For each mucus plug, a search was performed for the nearest airway termination point by Euclidean distance. Mucus plugs were then incorporated into the topological diagram of the airway tree. The lobe of each plug was assigned based on the lobe of the airway to which it localized, and the generation was computed by counting the number of airway bifurcations from the trachea, with the trachea considered generation 0.

### Estimation of RS.

To estimate the total effect of mucus plugs on airflow obstruction, we used the information generated in the airway mucus plug map for each CT scan to calculate a potentially novel measure of airway resistance (RS). First, the total airflow *Q* through the visible airway tree due to an applied pressure Δ*P* was computed by converting the airway tree into a network of resistive elements (Figure 6A, right). The length *L_n_* and radius *r_n_* of each airway segment *n* was used to estimate the resistance *R_n_* through that portion of the airway using *R_n_* = 8 μ *L_n_*/***π*** *r_n_*^4^, where μ is the dynamic viscosity of humidified air. The resulting series of flow and pressure equations at each node was then solved using previously published methods to obtain *Q* (24). Of note, the formula for *R_n_* reflects Poiseuille flow. Prior work has shown that, even if certain assumptions of Poiseuille flow are violated, the resistance of airway segments in the lung is still inversely proportional to the fourth power of airway radius (25). The effective resistance *R^a^* of the entire tree in the absence of mucus plugs was calculated as *R^a^* = Δ*P*/*Q^a^*. In the next step, we considered each terminal branch of the airway tree to be obstructed by associated mucus plugs, as determined in our airway mucus plug map. We recomputed *R^p^* as the net resistance with these branches blocked — i.e., flow is set at zero at those nodes. The RS was then calculated as the percentage increase in resistance due to plugs above the unplugged airway by RS = (100 × [*R^p^* – *R^a^*]/*R^a^*). We were unable to estimate RS in 3 of 97 scans (3%) because the processing pipeline did not converge on the parameter estimates for the entire airway. The Pearson correlation coefficient for *R^a^* between baseline and year-3 scans was 0.72.

### Estimation of OLVP.

After lobar segmentation, the voxels within each lobe were assigned to a specific airway branch by finding the nearest airway termination point, similar to ref. 26. Each subregion was then labeled as obstructed if a mucus plug occluded the terminal airway and unobstructed if a mucus plug was absent (Figure 6B). The OLVP for each lobe was estimated as the volume of voxels associated with an obstructed airway (V^o^) divided by the total voxel volume of the lobe (V^t^), or OLVP = (100 × V^o^/V^t^). The computation was performed on a lobar basis to ensure that lung parenchyma was not assigned to an airway branch opposite a fissure, after which the OLVP was then estimated for the entire lung. OLVP could not be computed in 3 of 97 scans (3%) where lobar segmentation failed.

### Measurement of regional air trapping.

Automated quantitative CT analysis was performed by Vida Diagnostics to estimate DPM air trapping, Jacobian mean, and LAA^856^% on a lobar level as previously described (10).

### Clinical survey data and physiologic measurements.

Clinical surveys of asthma control, comorbid conditions, spirometry, hematologic testing, and sputum characterization were collected and analyzed as part of the SARP-3 protocol (8, 15). Values were taken from the visit closest to the date of the designated CT scan. Not all patients had data for every study outcome, and analyses used available data.

### Statistics.

Statistical analyses were carried out using the SciPy, scikit-learn, and statsmodel packages in Python (27). Numeric nonparametric variables were evaluated by nonparametric methods including Kruskal-Wallis, Mann-Whitney *U*, or Wilcoxon signed-rank test (matched samples). Categorical variables were evaluated by χ^2^ analysis. Regression of numeric variables was quantified using the Spearman correlation coefficient (*r*_s_). For linear regressions on variables with repeated measurements from the same patient, *P* values were additionally confirmed using a linear mixed model with random effects for patient. A *P* value of less than 0.05 was considered significant. For analysis of proximal versus distal airway mucus plugs, confidence intervals for *r*_s_ of plug count by generation versus FEV_1_ and FEF_25–75_ were obtained by bootstrapping. In each bootstrapping sample, a set of 55 patients was generated using random resampling with replacement. The process was repeated 1,000 times. Statistical significance in comparing *r*_s_ for generation ≤ 7 and generation ≥ 10 was determined by estimating the 95 (*P* < 0.05) or 99 (*P* < 0.01) percentile value of the quantity (r_s_^gen≤7^ – r_s_^gen≥10^) from the bootstrap distribution. SHAP value analysis was carried out using the SHAP Python package (28). Directed acyclic graph analysis was performed using DAGitty (29).

### Study approval.

Written informed consent approved by each center’s IRB was received from participants prior to inclusion in the study. Study procedures and sample collection were carried out using standardized protocols approved by each center’s IRB.

### Data availability.

The Supporting Data Values file provides the values underlying the graphed data and the means reported in the main manuscript and in the supplement. The SARP-3 cohort database is available through dbGaP (https://www.ncbi.nlm.nih.gov/gap/) under the accession no. phs002788.v1.p1. Requests for access to lung images from participants in SARP-3 are considered by the SARP-3 steering committee on a case-by-case basis, and any such request can be facilitated by the corresponding author.

## Author contributions

BKH and JVF conceived of and designed the study, conducted the data analysis, and prepared the first draft of the manuscript. BME, TSH, LDH, and KGK annotated the CT lung scans for this study. MT, FH, CEM, NRB, SM, JC, SBF, EAH, MC, and PGW made substantial contributions to the design and analysis of the study. ATH, EI, NNJ, BDL, DTM, KS, and SEW are SARP investigators who reviewed the study proposal and participated in data discussions as data was being generated. All authors revised the draft critically for intellectual content.

## Supplementary Material

Supplemental data

Supplemental video 1

Supplemental video 2

Supplemental video 3

Supporting data values

## Figures and Tables

**Table 4:**
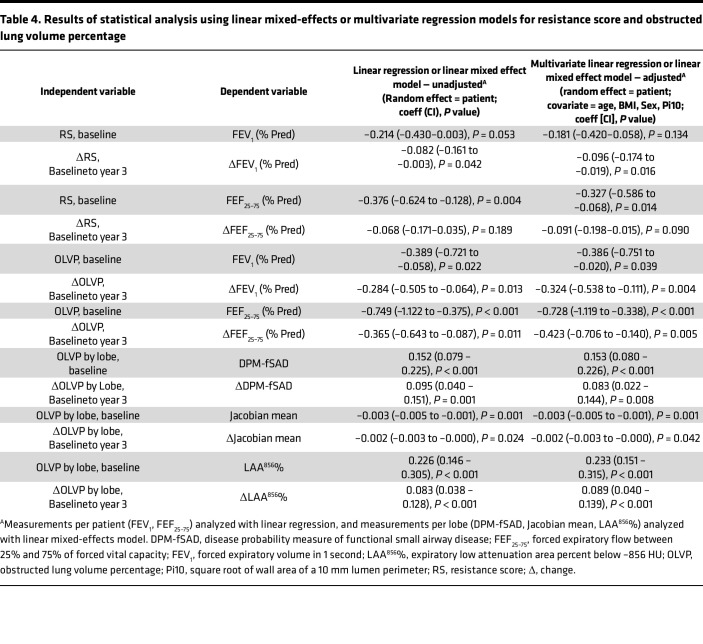
Results of statistical analysis using linear mixed-effects or multivariate regression models for resistance score and obstructed lung volume percentage

**Table 1:**
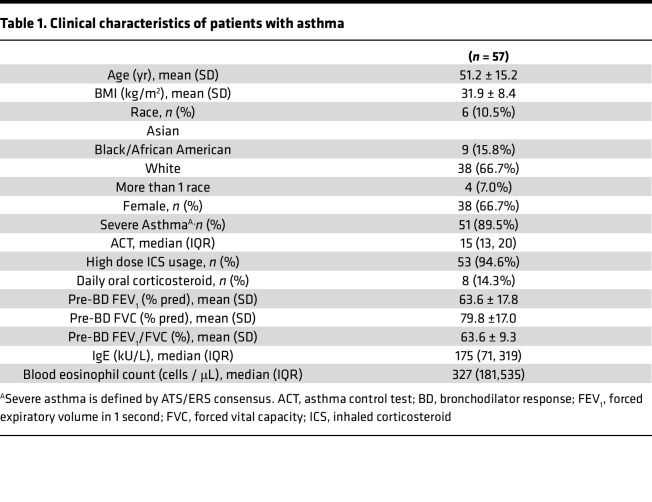
Clinical characteristics of patients with asthma

**Table 2:**
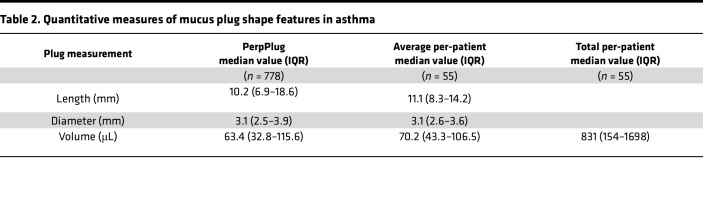
Quantitative measures of mucus plug shape features in asthma

**Table 3:**
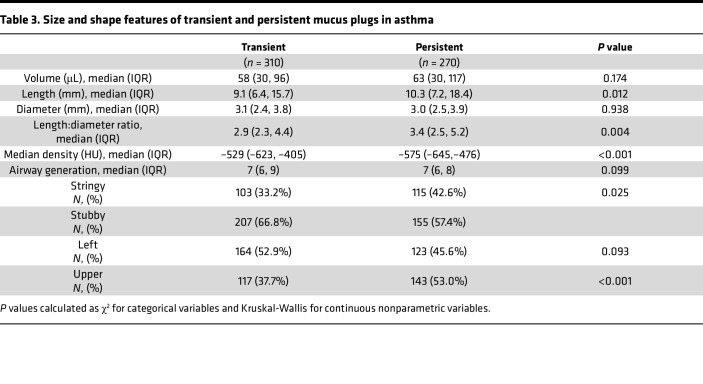
Size and shape features of transient and persistent mucus plugs in asthma

**Table 5:**
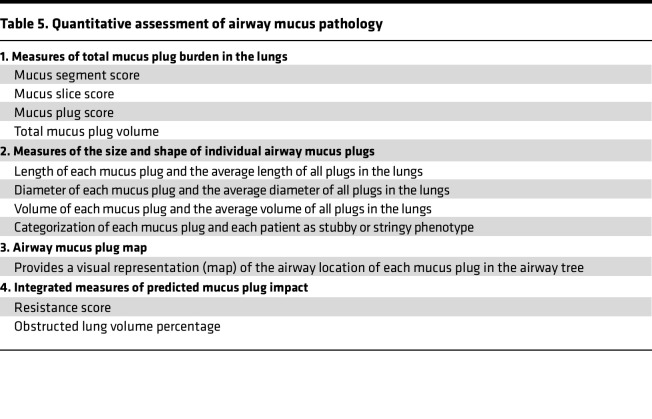
Quantitative assessment of airway mucus pathology

## Version 1 12/21/2023

In-Press Preview

## Version 2 02/08/2024

Electronic publication
