# Supplementary material for: Persistent mucus plugs in proximal airways are consequential for airflow limitation in asthma
Source: JCI Insight. 2024 Feb 8;9(3):e174124. doi: 10.1172/jci.insight.174124 (PMC10967478; doi:10.1172/jci.insight.174124)
Supplement: Supplemental data [file jciinsight-9-174124-s193.pdf]

Supplemental Materials

Persistent mucus plugs in proximal airways are consequential for airflow limitation in asthma

Authors and affiliations: Brendan K. Huang[1], Brett M. Elicker[2], Travis S. Henry[3], Kimberly G. Kallianos[2], Lewis D. Hahn[4], Monica Tang[1], Franklin Heng[5], Charles E. McCulloch[6], Nirav R. Bhakta[1], Sharmila Majumdar[2], Jiwoong Choi[7], Loren C. Denlinger[8], Sean B. Fain[9], Annette T. Hastie[10], Eric A. Hoffman[9], Elliot Israel[11], Nizar N. Jarjour[8], Bruce D. Levy[11], Dave T. Mauger[12], Kaharu Sumino[13], Sally E. Wenzel[14], Mario Castro[7], Prescott G. Woodruff[1,5], John V. Fahy[1,5] for the NHLBI Severe Asthma Research Program (SARP)

[1]Division of Pulmonary, Critical Care, Allergy and Sleep Medicine, Department of Medicine, University of California San Francisco, San Francisco, California;

[2]Department of Radiology and Biomedical Imaging, University of California San Francisco, California;

[3]Department of Radiology, Duke University, Durham, North Carolina;

[4]Department of Radiology, University of California San Diego, San Diego, California;

[5]Cardiovascular Research Institute, University of California San Francisco, San Francisco, California;

[6]Department of Epidemiology and Biostatistics, University of California San Francisco, San Francisco, California;

[7]Division of Pulmonary, Critical Care and Sleep Medicine, University of Kansas School of Medicine, Kansas City, Kansas;

[8]Division of Allergy, Pulmonary, and Critical Care Medicine, University of Wisconsin School of Medicine and Public Health, Madison, Wisconsin;

[9]Department of Radiology, University of Iowa, Iowa City, Iowa;

[10]Department of Internal Medicine, Section for Pulmonary, Critical Care, Allergy and Immunology, Wake Forest School of Medicine, Winston-Salem, North Carolina;

[11]Division of Pulmonary and Critical Care Medicine, Department of Medicine, Brigham and Women's Hospital, Boston, Massachusetts;

[12]Division of Biostatistics and Bioinformatics, Penn State College of Medicine, The Pennsylvania State University, Hershey, Pennsylvania;

[13]Division of Pulmonary and Critical Care Medicine, Washington University, St. Louis;

[14]Department of Environmental and Occupational Health, University of Pittsburgh, Pittsburgh, Pennsylvania;

Correspondence and requests for reprints should be addressed to John V. Fahy, M.D., Room 1307, Health Sciences East, University of California San Francisco, San Francisco, CA 94143-0130. E-mail: john.fahy@ucsf.edu.

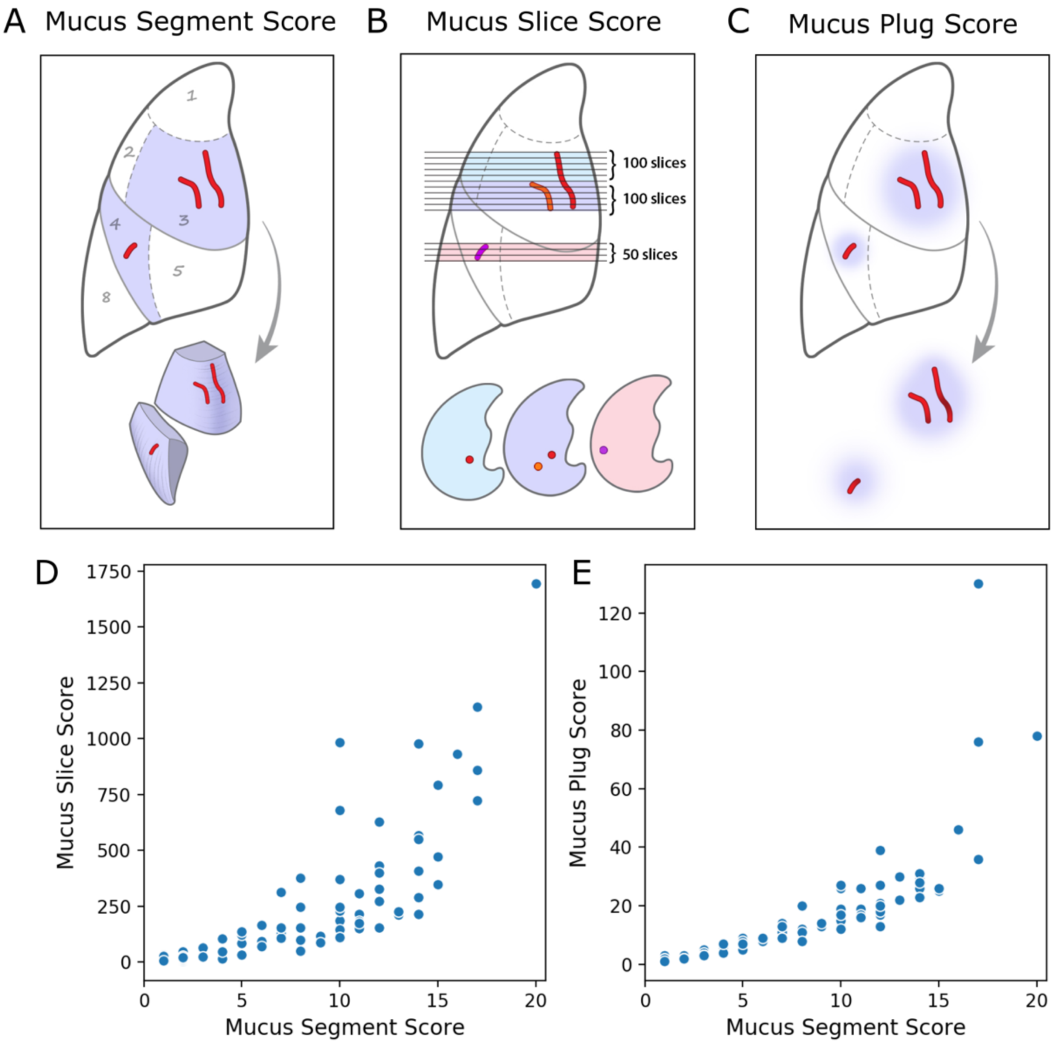

Supplemental Figure S1. The mucus slice score and mucus plug score are two new measures of total mucus plug burden.
In comparison to the previously described **(A)** mucus segment score, **(B)** the mucus slice score and **(C)** mucus plug score are two new measures of mucus plug burden. In the example illustrated, three mucus plugs are located in two different bronchopulmonary segments. The segment score is 2, the slice score is 100 x 1 + 100 x 2 + 50 x 1 = 350, and plug score is 3. **(D)** Comparison of mucus segment score vs slice score for all scans with mucus plugs (n=97) **(E)** Comparison of mucus segment score vs mucus plug score for same group.

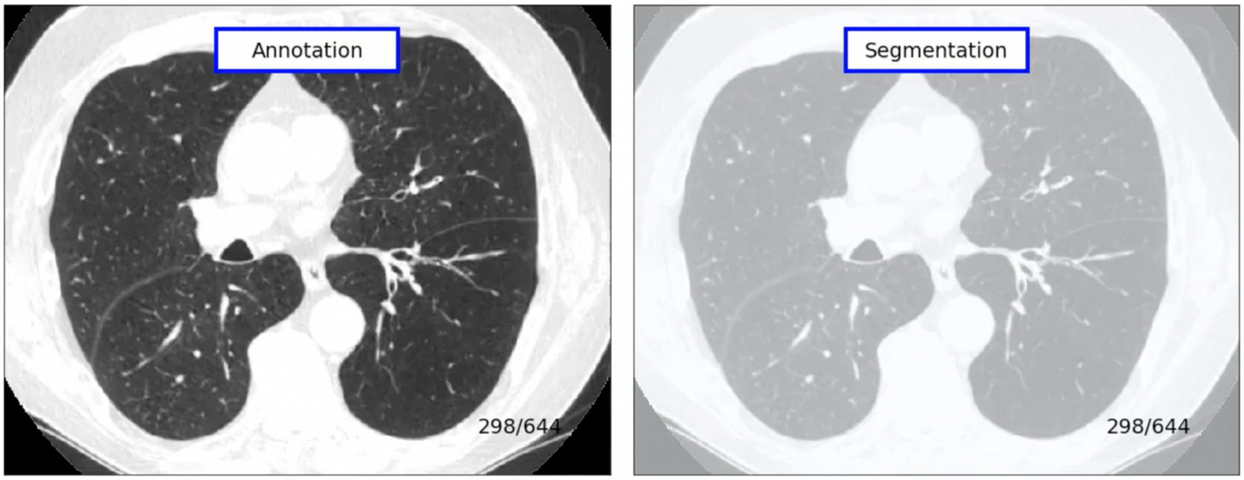

Supplemental Movie S1. Example of plug annotation and segmentation.

(Left) Example annotation of mucus plugs in a CT volume with individual plugs being labelled by number with an elliptical marker. (Right) Results of voxel-wise segmentation.

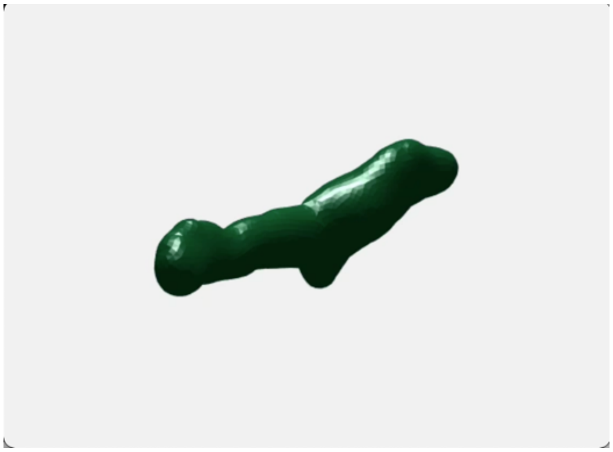

Supplemental Movie S2. Three-dimensional rendering of selected plugs

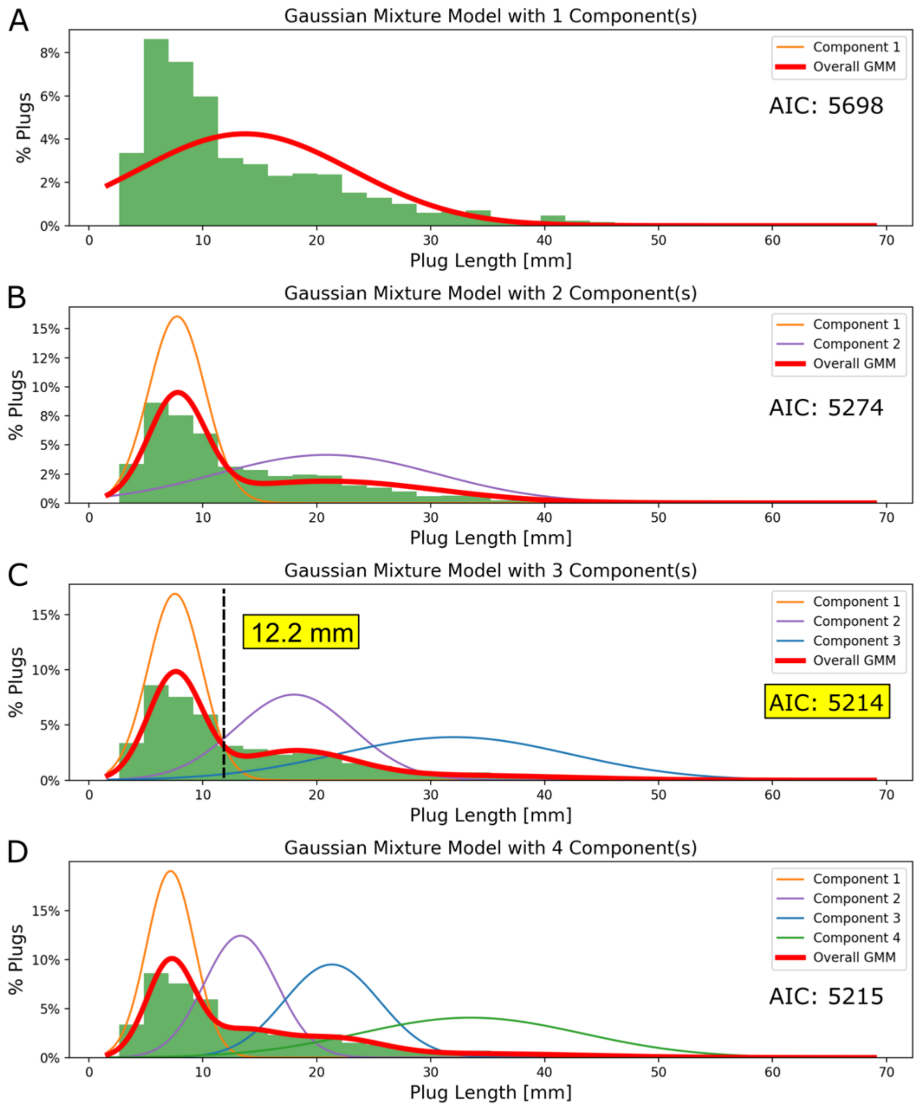

Supplemental Figure S2. Model selection for plug length distribution.

Histogram of plug lengths (n=778) with best fit Gaussian mixture model consisting of **(A)** one, **(B)** two, **(C)** three, and **(D)** four underlying Gaussian components. Akaike information criterion (AIC) is defined as AIC = 2k – 2 ln(L), where k is the number of model parameters and ln(L) is the log-likelihood function for each individual model. The three-component model **(C)** has minimal AIC, with classification boundary between component 1 and component 2 of 12.2 mm determined by point of equal probability of weighted components. Note that Gaussian components graphed in this supplemental figure are normalized to unity for optimal visualization.

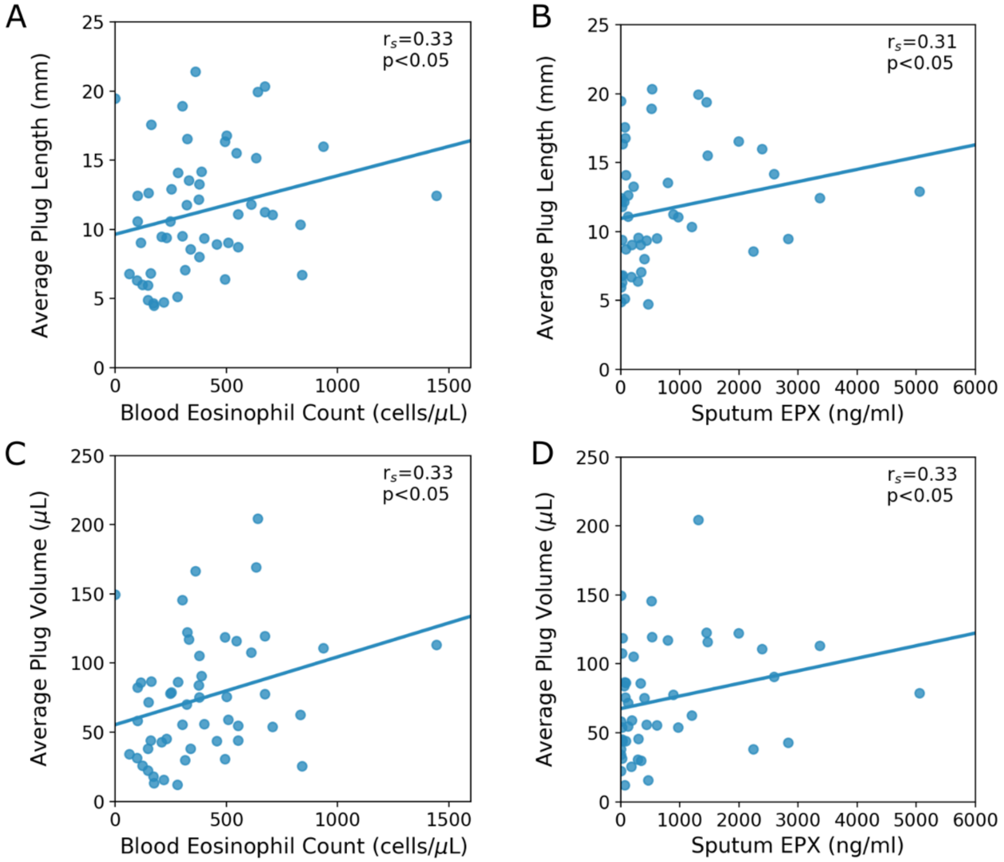

Supplemental Figure S3. Longer and larger plugs are associated with higher blood eosinophil counts and sputum eosinophil peroxidase levels.

(A) Correlation of blood eosinophil count (n=52) and **(B)** sputum eosinophil peroxidase (EPX) (n=46) with average plug length per patient. **(C)** Correlation of blood eosinophil count (n=52) and **(D)** EPX (D, n=46) with average plug volume per patient. r_s denotes Spearman correlation coefficient.

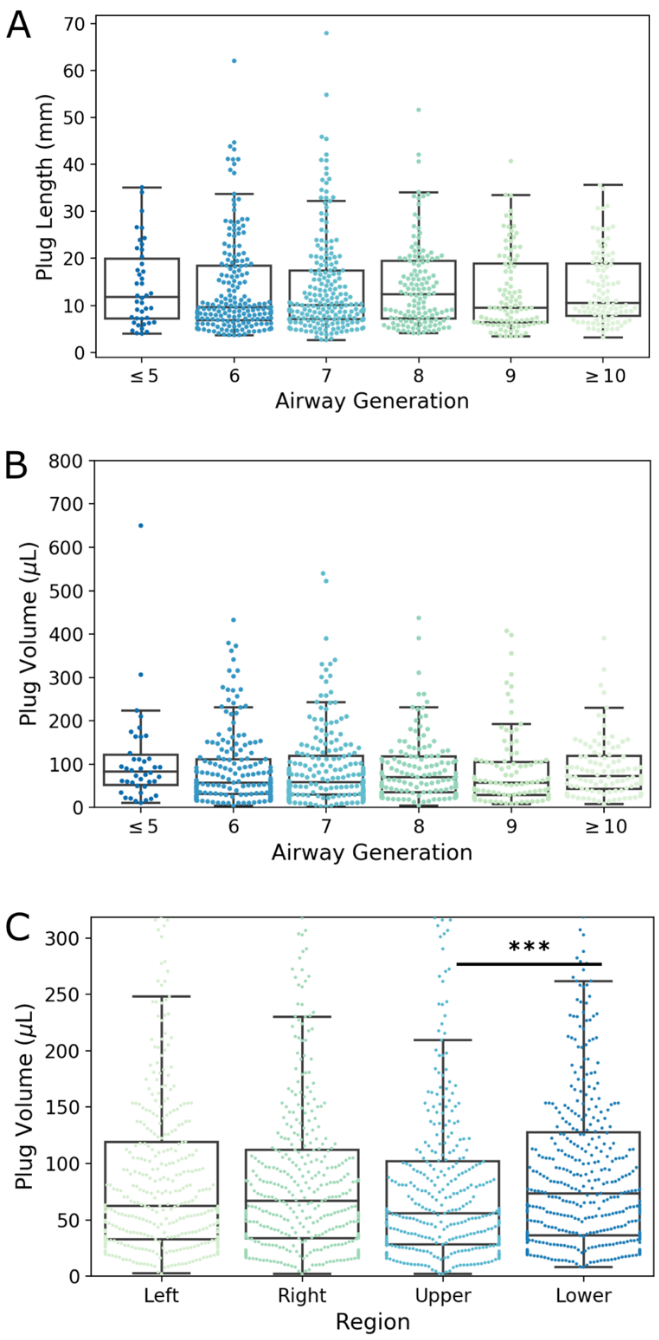

Supplemental Figure S4. Proximal mucus plugs are similar length and volume to distal plugs, while lower lobe mucus plugs are larger on average than upper lobe plugs.

(A) Distribution of mucus plug length and **(B)** volume for baseline cohort of patients (n=778 plugs) grouped by airway generation. Regression analysis not statistically significant by Spearman correlation coefficient. **(C)** Distribution of plug volume by region of lung, with upper region including both upper lobe and middle lobe. *** Indicates significant difference from upper lobe, $p<0.001$ by Mann-Whitney U Test.

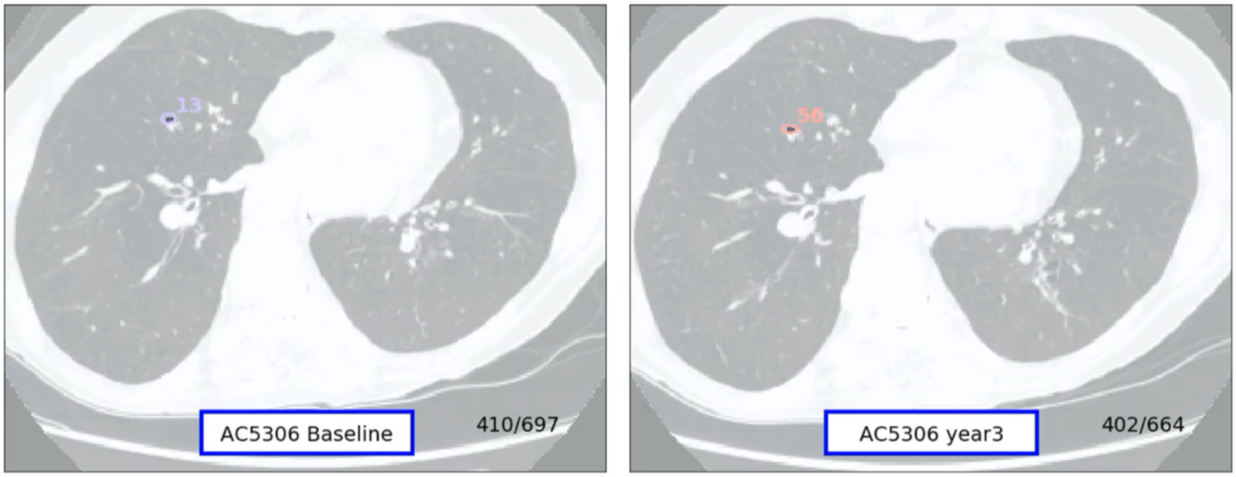

Supplemental Movie S3. Example of persistent plug in the same airway at baseline and year 3.

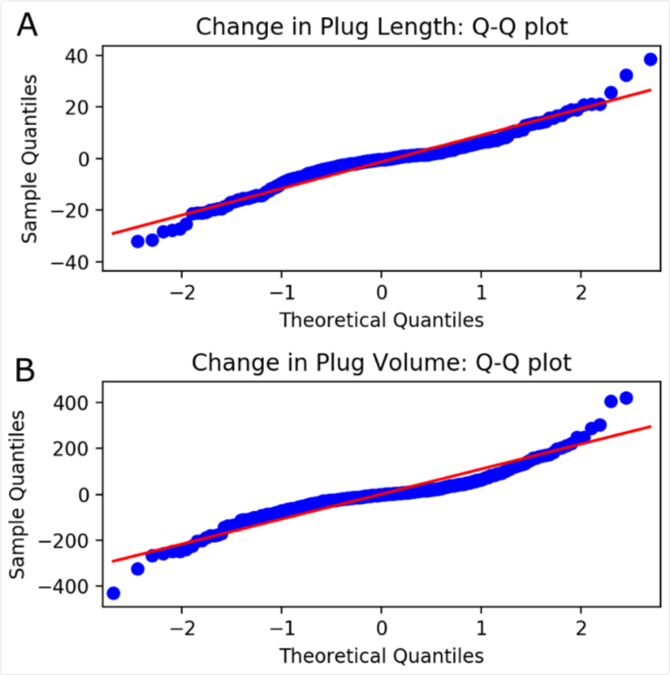

Supplemental Figure S5. Changes in length and volume over three years approximate normal distribution.

(A) Quantile-quantile (Q-Q Plot) showing changes in length (mm, blue dots) of persistent plugs (n=270) between baseline and year 3 scan, compared to theoretical normal distribution (red line). **(B)** Q-Q Plot showing changes in volume (μL, blue dots) of persistent plugs (n=270) between baseline and year 3 scan, compared to theoretical normal distribution (red line).

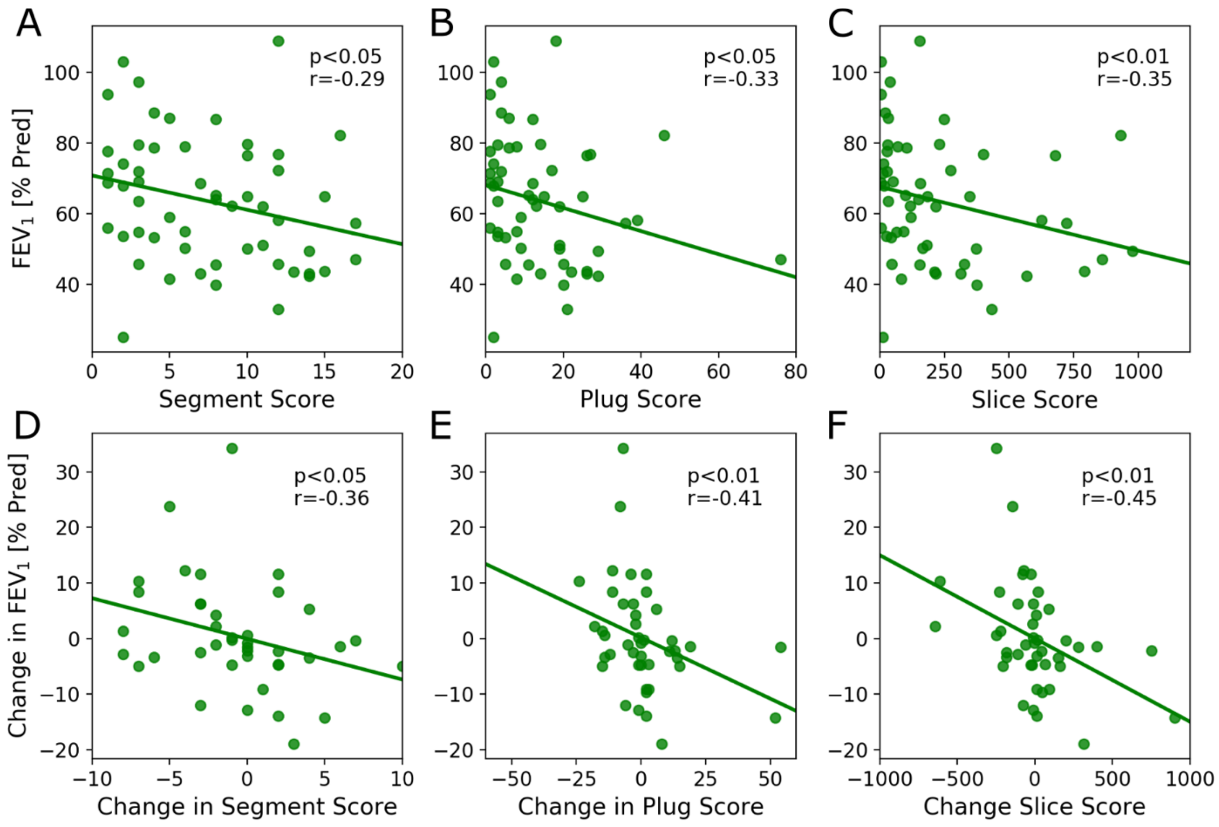

Supplemental Figure S6. The mucus segment score, plug score, and slice score are associated with worse airflow obstruction.

Relationship between **(A)** mucus segment score, **(B)** mucus plug score, and **(C)** mucus slice score and forced expiratory volume in 1 second (FEV_1) amongst patients at baseline (n= 55). Relationship between longitudinal changes in **(D)** mucus segment score, **(E)** mucus plug score, and **(F)** mucus slice score with changes in FEV_1 across three years (n= 43). r denotes Spearman coefficient.

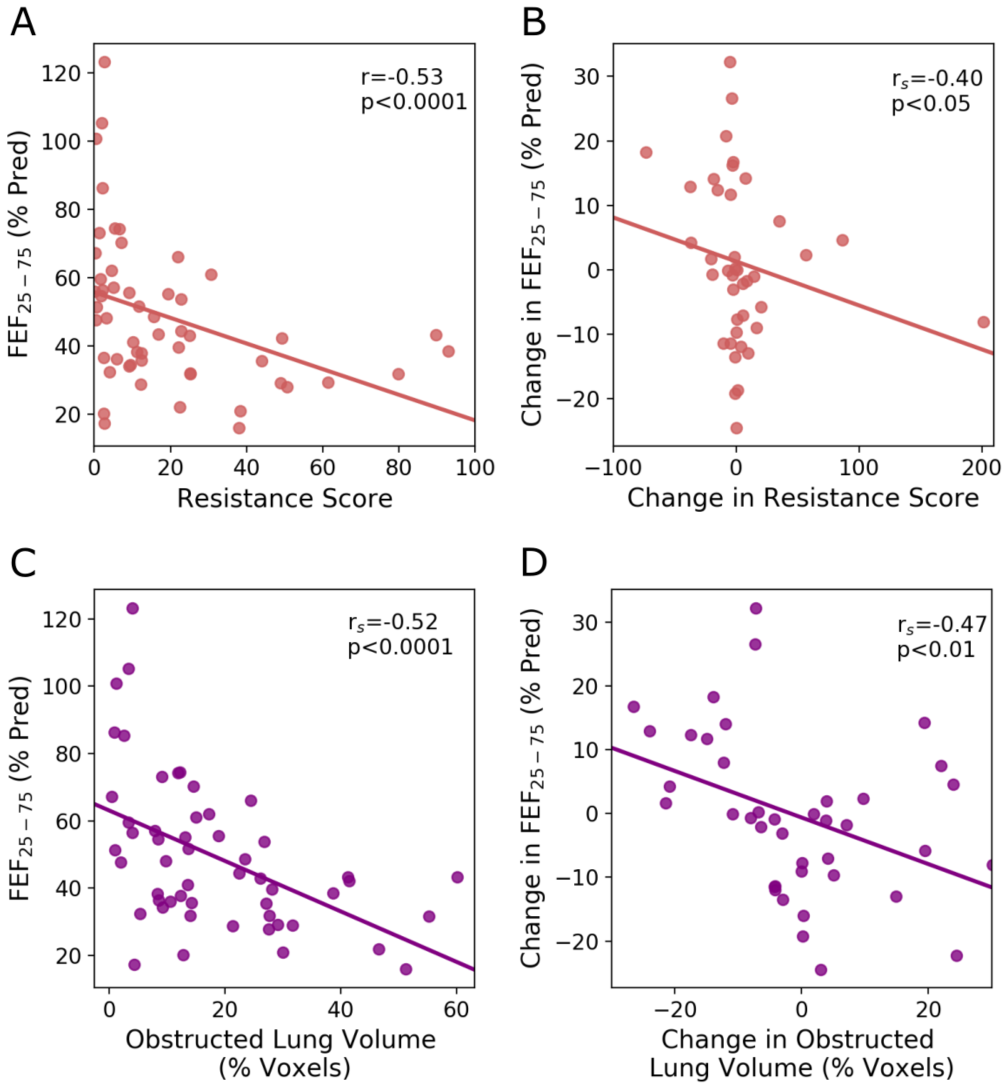

Supplemental Figure S7. The resistance score and obstructed lung volume percentage are inversely associated with measures of small airways function.

(A) Relationship between resistance score and forced expiratory flow between 25% and 75% of forced vital capacity ($FEF_{25\text{-}75}$) at baseline (n=54) and **(B)** comparing longitudinal changes across three years (n=40). **(C)** Relationship between obstructed lung volume percentage and $FEF_{25\text{-}75}$ at baseline (n=53) and **(D)** comparing longitudinal changes across 3 years (n=40). r_s denotes Spearman coefficient.

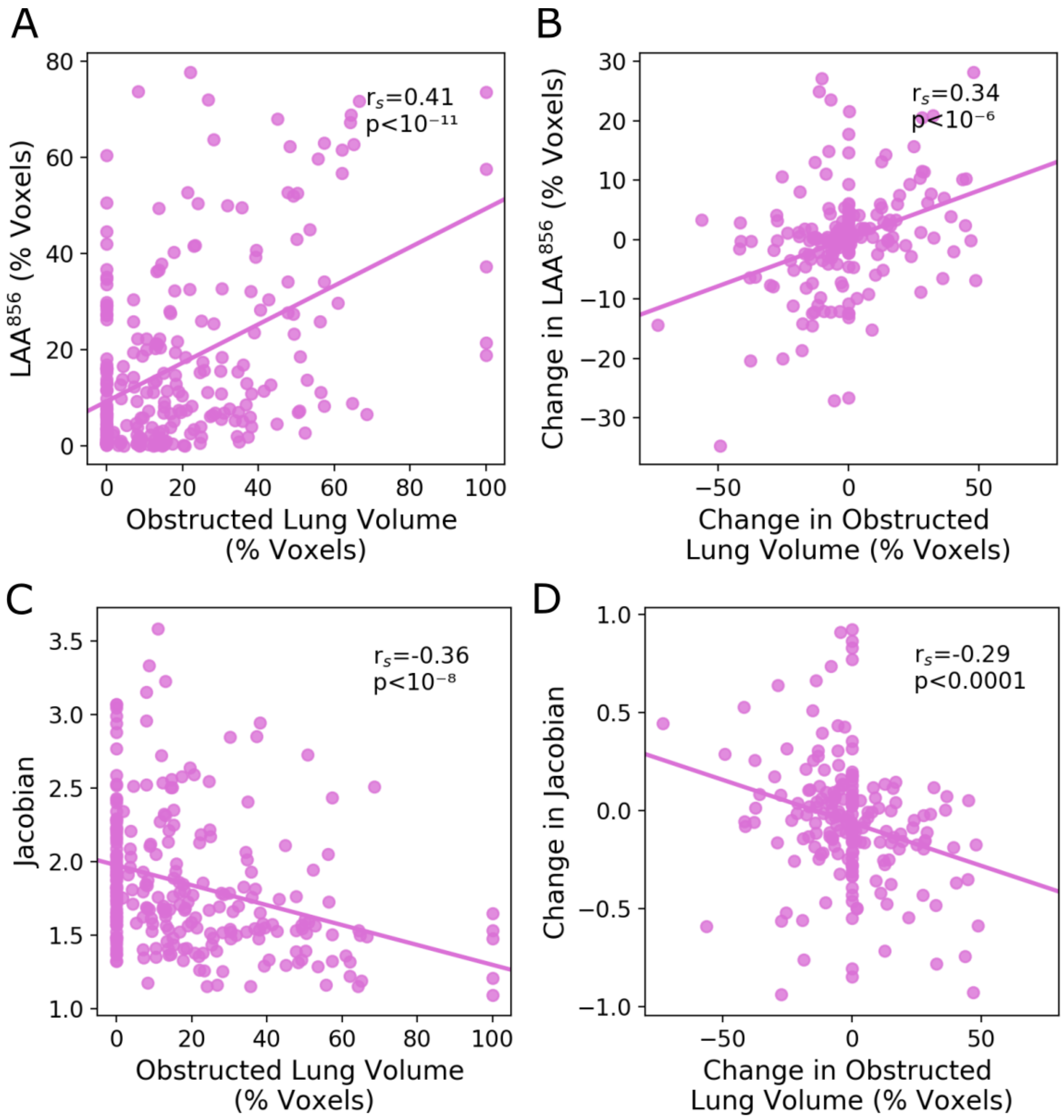

Supplemental Figure S8. The obstructed lung volume percentage is spatially associated with additional measures of air trapping.

(A) Relationship between obstructed lung volume percentage (OLVP) per lobe and expiratory low attenuation area percent below -856 HU (LAA^{856}%) at baseline (n=260) and **(B)** comparing longitudinal changes across three years (n=195). **(C)** Relationship between OLVP per lobe and Jacobian mean at baseline (n=260) and **(D)** comparing longitudinal changes across 3 years (n=195). r_s denotes Spearman coefficient.

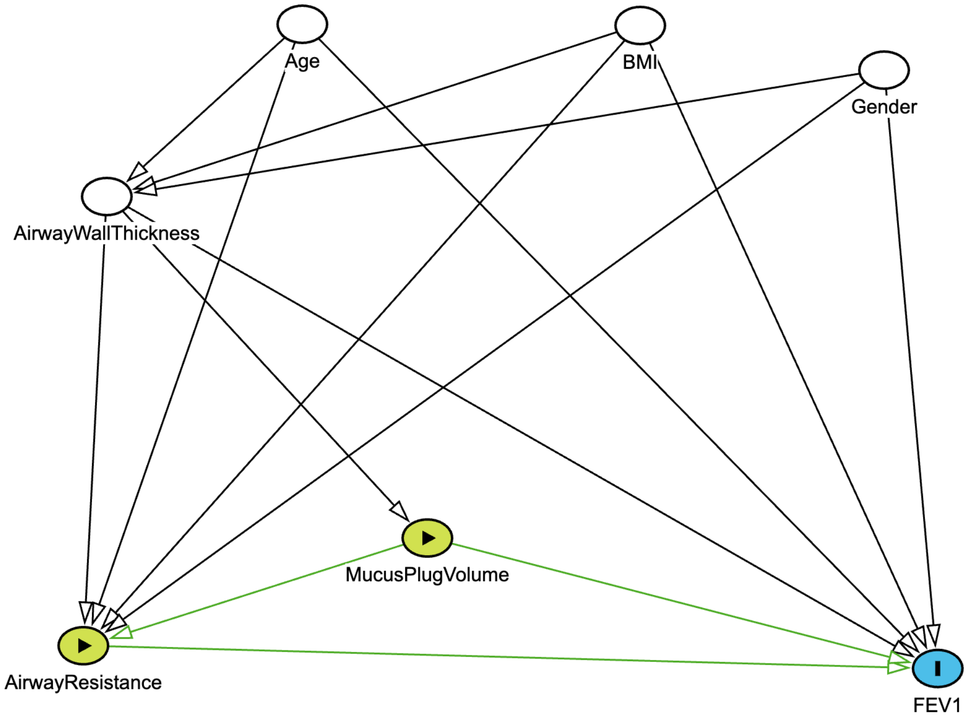

Supplemental Figure S9. Directed acyclic graph

Directed acyclic graph showing hypothesized causal relationships. Minimal sufficient adjustment set is found to be age, BMI, gender, and airway wall thickness (measured as Pi10 - square root of wall area of a 10-mm lumen perimeter).

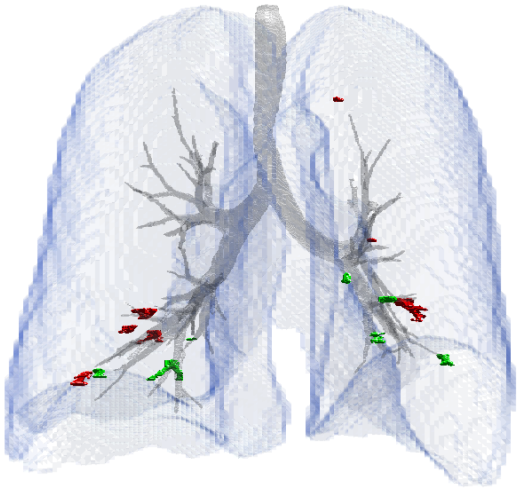

Supplemental Figure S10. Example rendering of persistent and new plugs at a follow up scan.

Rendering of follow up scan showing segmented lung parenchyma (blue), airways (grey), and persistent (present at baseline and follow up scan, red) versus new (present at follow up but not at baseline, green) mucus plugs in a patient with asthma.
